# Team Sports Off the Field: Competing Excludes Cooperating for Individual but Not for Team Athletes

**DOI:** 10.3389/fpsyg.2019.02470

**Published:** 2019-11-05

**Authors:** Florian Landkammer, Kevin Winter, Ansgar Thiel, Kai Sassenberg

**Affiliations:** ^1^Leibniz-Institut für Wissensmedien, Tübingen, Germany; ^2^University of Tübingen, Tübingen, Germany

**Keywords:** competition and cooperation, co-opetition, team athletes and interindividual differences, information sharing, carry-over effect

## Abstract

Both team and individual sports require competition, whereas cooperation is more prevalent in team than in individual sports. In particular, team athletes have to compete (for starting roles) while cooperating (for team success) with the same teammates. For team athletes, competition and cooperative behavior, two mutually exclusive constructs according to earlier psychological research, might therefore be less incompatible than for individual athletes. In Study 1, team athletes attributed a higher demand to compete and cooperate with the same teammates or training partners to their sport than individual athletes to their sport. Study 2 showed that experiencing competition (vs. control) undermines information sharing less for team than for individual athletes. In addition, Study 2 demonstrated that priming competition undermines the accessibility of cooperative thoughts less for team than for individual athletes. Therefore, team athletes might be better at competing without ceasing to cooperate. Implications for collaboration in groups are discussed.

## Introduction

The distinction between team and individual athletes is a key classification in sports (e.g., Allen et al., 2011). Yet, Allen et al. (2013) noted that after nearly 70 years of research relatively little is known about inter-individual differences between team and individual athletes. In their review on the relation between sports and personality, they concluded that team athletes tend to be more extraverted and less conscientious than individual athletes. No clear differences were found in social skills. We believe that the missing association between sports affiliation and social skills might result from the focus on broad skills and personality characteristics in earlier research. Therefore, we aimed to apply a more fine-grained approach similar to a person x situation interaction suggested by Mischel and Shoda (2010; see also Shoda and Mischel, 2000): By testing the response of team versus individual athletes to competition (vs. control), we aimed to target a specific difference between those two groups regarding their social behavior.

In general, individuals who engage in competitive sports practice with other athletes. This not only applies to team sports, but also to individual sports (Evans et al., 2012). Thus, athletes are usually situated in social environments when training for tournaments or matches (cf., Martin et al., 2014). In addition, athletes rely on each other (i.e., are positively interdependent; Deutsch, 1949) when competing as a group against other groups, for example during soccer games or relay swimming competitions. In fact, competition (i.e., negative interdependence; Deutsch, 1949) between athletes or teams can be seen as a fundamental characteristic of the social context of – as the name implies – competitive team as well as individual sports.

Competing individuals usually do not show cooperative behavior toward each other during the competitive situation (Johnson and Johnson, 1989), because such behavior would undermine the likelihood of their own success (Campbell, 1965). Yet, in real world social contexts, pure competition rarely occurs (Deutsch, 1949). For instance, sport competitions require at least a minimum level of cooperation among the competitors in following of common rules (Lüschen and Weis, 1976). In some contexts, individuals must even explicitly compete and cooperate with the same interaction partners. This has been labeled *co-opetition* (Galinsky and Schweitzer, 2015; Landkammer and Sassenberg, 2016). In psychological terms, co-opetition is a social context characterized by so-called mixed-motive interdependence (e.g., De Dreu et al., 2008). However, other than in the social dilemmas usually studied in the mixed-motive interdependence literature, co-opetition does not allow to opt for either competing *or* cooperating. Instead, individuals situated in co-opetition have to reconcile both the demand to compete *and* to cooperate with one and the same person (s; Landkammer and Sassenberg, 2016).

Despite many similarities between team and individual sports introduced above, co-opetition is fundamentally more prevalent in team sports. On the one hand, team athletes have to permanently compete with their teammates (e.g., for starting roles and other status-related resources). One can imagine that officials or coaches might even try to foster such inner-team competitions hoping for performance enhancements of their players. On the other hand, team athletes also have to continually cooperate with their teammates during the performance in order to accomplish the task, improve as a team and to win against other teams. Cooperation in this sense not only means having a joint goal; it also includes behavioral interdependence (i.e., showing behavior that enables other athletes to perform well, e.g., passing the ball that allows a teammate to score a goal). In the current research, accumulating individual performances to a joint “team score”, like in relay competitions or track and field events, is therefore distinguished from team sports. In this case, individual athletes have a joint goal, but they are able to perform without any behavioral interactions with their teammates. Team sports, thus, require competing *and* cooperating with the *same others* to a large extent *while performing* (cf., Rees and Segal, 1984; Van Yperen, 1992). Same others, here, refers to the same teammates or training partners. When opting to exclusively compete with teammates, the team as a whole could not prevail over other teams and the player would probably not be a member of the team for very long. When opting to cooperate without asserting oneself (e.g., without demonstrating superiority in scoring goals), the athlete is unlikely to get a starting position or an important role within the team. Thus, team athletes are frequently situated in co-opetition.

In individual sports, this simultaneous demand to compete and to cooperate *while performing* is less pronounced. Even though in the context of elite sport, individual athletes have to behave cooperatively *in general* to ensure they work well with their support team, cooperative behavior *while performing* is less important than in team sports. In fact, most of the time individual sports exclusively require athletes to outperform others during practice or tournaments. Even in relay competitions (e.g., in swimming), individual performances are aggregated to a team performance, while no cooperative behavior within the team is required during the competition. This means that swimmers of the same team might support each other motivationally (e.g., by cheering) for a joint goal. However, a swimmer does not explicitly rely on the cooperative action of another athlete that would enable him or her to perform the task (e.g., to perform freestyle movements). In contrast, a soccer player is behaviorally interdependent of his teammates and is, for example, not able to score a goal without other team players passing the ball to him or her. Taking this into consideration, relay competitions do not contain behavioral interdependence and are not regarded as team sports here.

As a consequence of co-opetition being an inherent part of team sports, team athletes should represent the relationship between competition and cooperation differently from others, including individual athletes. Representing competition usually undermines cooperative teamwork, for instance, in the context of joint decision making in organizations or politics (for a review, see Wittenbaum et al., 2004). In particular, competition among group members has a negative impact on sharing task-relevant information. This has been shown for individuals (a) competing for a higher status (Ray et al., 2013; Toma et al., 2013), (b) trying to maximize their own outcomes (Steinel and De Dreu, 2004) and (c) competing for being the first to know the right decision (Toma and Butera, 2009; Steinel et al., 2010). On a cognitive level, these effects can be explained by a representation of competition that excludes or inhibits cooperative thoughts and behavior: When encoding a situation as competitive, individuals rely on this representation and cease cooperative *behavior* and *thinking* (i.e., they apply a competition mindset; see Sassenberg et al., 2007). In other words, competition should *spontaneously reduce cooperative thoughts and behavior*. In line with this reasoning, situations demanding to compete and to cooperate with same others (i.e., co-opetition) entails conflicting demands – and the need to reconcile two demands that initially are in conflict makes individuals more flexible in subsequent tasks (Landkammer and Sassenberg, 2016).

The inherent need to reconcile both competitive and cooperative demands in co-opetition and thus in team sport is likely to co-occur with a less inhibitory relationship between competition and cooperation. As a consequence, team athletes might be better at behaving and thinking cooperatively when experiencing competition. In other words, team athletes’ cognitive representation of competition should exclude cooperating to a lesser extent than individual athletes’ representation of competition. This might be due to a self-selection of team athletes to their sport or because of certain socialization within team sports over time. In sum, this leads us to the following hypotheses: Because co-opetition seems to be particularly present in team sports, we predict that team athletes perceive a higher demand to compete and to cooperate with the same others (i.e., co-opetition) during performance than individual athletes (Hypothesis 1). These “others” are teammates within team sports, whereas in individual sports “others” rather refer to training partners. Study 1 tested this basic assumption about the demands experienced by team and individual athletes. We further predicted that for individual athletes, competition (vs. control) subsequently reduces cooperative behavior in an unrelated task (*here*: information sharing), whereas this carry-over effect of competition should be weaker for team athletes (Hypothesis 2). Moreover, semantically priming competition via a lexical decision task (LDT) should reduce the accessibility of cooperative thoughts in individual athletes, but less so in team athletes (Hypothesis 3).

Ethical approval for the current studies was provided by the institutional review board (Lokale Ethikkommission) of the Leibniz-Institut für Wissensmedien (Tübingen, Germany; LEK 2011/013). The inclusion of the non-adult sample in Study 2 was additionally approved by the Olympic Sports Center Rhein-Neckar (Heidelberg, Germany). Written informed consent was obtained from all adult participants as well as from the respective coaches of the Olympic Sports Center (OSP). According to German law the OSP was able to provide (and provided) informed consent for the youth athletes as it has supervision duty.

## Study 1

In Study 1, we asked team and individual athletes from different types of sports to indicate the degree to which they experience simultaneity of competitive and cooperative demands in their focal sport. We predicted that team athletes should perceive a higher degree of simultaneity of competitive and cooperative demands in their sports compared to individual athletes (Hypothesis 1). To replicate earlier findings that team (compared to individual) athletes are more extraverted and less conscientious (see Allen et al., 2013), we also included a measure of the big five. We did not follow a specific research question by adding this measure, but wanted to gather data for possible meta-analyses. Beyond this, there hardly seem to be any differences between team and individual athletes in social skills, particularly in aspects of personality that relate to competition or cooperation (see also Kanning and Kappelhoff, 2012). To *rule out* the existence of such differences in our sample – which would be a confounding factor in the subsequent experiments – this study additionally included goals and values related to competition (i.e., social value orientation, social comparison orientation, and achievement goals).

### Method

#### Participants

In order to recruit athletes, data collection was scheduled for a specific time frame at a university sports facility, in which we aimed to recruit as many participants from this specific sample as possible.

One hundred and four students (40 women, *M*_age_ = 22.53, range = 18–33 years; one participant did not provide demographics) with a major in sports science participated in return for a bar of chocolate and a piece of fruit. After signing an informed consent, participants indicated, whether they *predominantly* engaged in team or individual sports (i.e., their focal sport). Fifty-nine indicated that they frequently do team sports (33 soccer, 11 handball, 6 basketball, 5 volleyball, and 4 other team sports that were just mentioned by one individual) and 44 stated that they engaged in individual sports (15 athletic sports/running, 9 gymnastics, 4 tennis, 3 swimming, 3 climbing, 3 skiing, and a number of other sports just mentioned by one or two people). One participant was excluded from the analysis because s/he did not indicate sports affiliation. There were no differences between both athlete groups concerning the years they had engaged in their sport, *F*(1, 96^1^) = 0.07, *p* = 0.790, or the times they practice together with other athletes, *F*(1, 96) = 0.24, *p* = 0.625. On average, these athletes engaged in their focal (team or individual) sport for about 12.5 years (*SD* = 5.51), practiced around three times a week (*SD* = 1.43), *regularly* together with others (i.e., 97% of team and individual athletes practiced together with others in the majority of their training sessions). Thus, *both* athlete groups engaged in formal-competitive rather than self-organized leisure sports and usually practiced together with other athletes.

#### Demand to Compete While Cooperating

Given that the demand to compete while cooperating has not received any attention in earlier research, we created a new scale. In order to test whether team athletes attribute a higher demand to compete and to cooperate with same others to their focal sport than individual athletes do, all participants responded to 10 items related to their practice or sport in general (original items in German; examples for the English translation: “In order to be successful at my sport, you often have to prevail against the same people you cooperate with.”; “In my sport it is important to demonstrate your superiority in certain areas in comparison to others with whom you share the same goal.”, α = 0.77) on a scale ranging from 1 (*does not apply*) to 7 (*applies completely*). We conducted a principle component analysis across these 10 items. The Kaiser criterion clearly indicated that a one factor solution is appropriate. The factor explains 35% of all item’s variance. All items loaded >0.35 [0.377; 0.728] on this factor. Therefore, the items were summarized into a single scale (α = 0.77). The internal consistency could not be improved by removing any of the items. Please see Appendix for the full item list.

#### Chronic Differences Related to Competition and Cooperation

We also assessed several personality measures related to competition to rule out potential differences between team and individual athletes that could have affected the perceived demand to compete while cooperating. *Social value orientation* (Van Lange, 1999) was assessed in 9 decomposed games with three options each. Following Van Lange (1999), athletes were classified in three orientations if at least 6 choices were consistent with one of them, resulting in 15 competitors, 45 prosocials, and 27 individualists. Besides that, measures for *social comparison orientation* (Gibbons and Buunk, 1999; German translation: Jonas and Huguet, 2008; 12 items, α = 0.73, scale from 1 = *not true* to 6 = *completely true*) and *achievement goals* (Elliot and Murayama, 2008; subscales with three items each: performance approach, α = 0.84; performance avoidance, α = 0.78; mastery approach, α = 0.74; mastery avoidance, α = 0.57; scale from 1 = *does not apply* to 7 = *completely applies*) were applied. Five additional self-developed items assessed perceptions of cooperation and competition in work environments. These items did not form a scale with adequate internal consistency (α = 0.47) and were, not included in the analysis.

#### Personality

We assessed the *big five* with a brief questionnaire (Rammstedt and John, 2005; subscales: extraversion, 4 items, α = 0.70; agreeableness, 4 items, α = 0.69; conscientiousness, 4 items, α = 0.71; neuroticism, 4 items, α = 0.70; openness, 5 items, α = 0.71; scale from 1 = *very inapplicable* to 5 = *very applicable*; see Appendix for the items). Given that Rammstedt and John (2005) report mean correlations of these short-scales with full scales of 0.85–0.91, the short scales used here seem to capture the big five without a substantial loss in measurement quality.

#### Data Analysis

Prior to the main data analysis, all indicators were screened for skewness and deviation from normal distribution was tested. No evidence for skewness was found (| skewness| <0.7). According to the Shapiro–Wilk test for normal distribution, only the mastery approach scale was not normally distributed (*p* < 0.001; all other *p* > 0.05/number of scales). We applied parametric tests to all measures and added a non-parametric test (Mann–Whitney-Test) to analyze mastery approach goals.

To be more precise, we compared (a) the demand to compete while cooperating (test of Hypothesis 1), (b) chronic differences related to competition and cooperation except for social value orientation, and (c) differences in personality depending on sports affiliation using a one-way analyses of variance (ANOVA) with sports affiliation as independent variable and the respective other measure as dependent variable. For social value orientation, we conducted a χ^2^ test for a 2 (sports affiliation) × 3 (social value orientation) table. To rule out that differences in demand to compete while cooperating were redundant with chronic differences related to cooperation and competition or differences in personality, we conducted an analysis of covariance (ANCOVA). Therefore, we tested the relation between sports affiliation (independent variable) and the demand to compete while cooperating (dependent variable), controlling for the person characteristics that differed between the two types of sports affiliations (covariate).

### Results

#### Demand to Compete While Cooperating

In line with Hypothesis 1, an ANOVA with sports affiliation (team vs. individual) as between-subjects factor and the demand to compete while cooperating as dependent measure revealed that team athletes, indeed, attribute a higher demand to their sport than individual athletes do, *F*(1, 101) = 18.11, *p* < 0.001, η*^2^_*part*_*_._ = 0.152, *MD* = 0.72, CI_95__%_ = [0.380, 1.045] (see Table 1 for descriptive statistics).

**TABLE 1:** Means and standard deviations for team and individual athletes from the Study 1 (*N* = 103).

	**Team**		**Individual**	
	**athletes**		**athletes**	
	Mean/*N*	SD	Mean/*N*	SD
Demand to compete while cooperating	5.46^∗^	0.81	4.74^∗^	0.87
**Social value orientations**				
Competitors	*N* = 5		*N* = 10	
Prosocials	*N* = 22		*N* = 23	
Individualists	*N* = 10		*N* = 17	
Social comparison orientation	3.91	0.53	4.07	0.64
**Achievement goals**				
Performance approach	4.46	1.23	4.22	1.48
Performance avoidance	4.79	1.01	4.39	1.39
Mastery approach	4.92^∗^	1.15	5.46^∗^	1.15
Mastery avoidance	4.51	1.09	4.63	1.41
**Big5**				
Extraversion	3.91	0.66	3.91	0.68
Agreeableness	3.42	0.71	3.22	0.82
Conscientiousness	3.41^∗^	0.76	3.89^∗^	0.52
Neuroticism	2.55	0.65	2.80	0.86
Openness	3.53	0.72	3.70	0.65

*^∗^Indicate significant differences.*

#### Chronic Differences Related to Competition and Cooperation

There were no differences between team and individual athletes regarding the distribution of the three social value orientations, χ^2^ (2, *N* = 103) = 1.60, *p* > 0.250, social comparison orientation, *F*(1, 101) = 2.04, *p* = 0.157, η*^2^_*part*_*_._ = 0.020, chronic performance approach or the mastery avoidance goals, both *F*s < 1.0. There was a trend for team athletes to indicate more performance avoidance goals than individual athletes, *F*(1, 101) = 2.93, *p* = 0.090, η*^2^_*part*_*_._ = 0.028. Also, team athletes had less strong mastery approach goals than individual athletes, *F*(1, 101) = 5.26, *p* = 0.024, η*^2^_*part*_*_._ = 0.050, Mann–Whitney-Test: *U* = 950, *z* = 2.33, *p* = 0.020.

#### Personality

Finally, we did not find differences in four of the big-five subscales, all *F*s < 2.7, *p*s > 0.1, η*^2^_*part*_*_._ < 0.15. However, in line with Allen et al. (2013), team athletes reported less conscientiousness than individual athletes, *F*(1, 101) = 13.01, *p* < 0.001, η*^2^_*part*_*_._ = 0.114.

#### Additional Analysis

We additionally wanted to test whether the difference between participants with individual and team sports affiliation on the demand to compete while cooperating remains significant after controlling for mastery approach goals and conscientiousness (the two person characteristics that differed depending on sports affiliation). Therefore, we conducted an ANCOVA with sports affiliation as independent factor, demand to compete while cooperating as dependent variable and mastery approach goals and conscientiousness as covariates. The effects of sports affiliation on the demand to compete while cooperating remained significant in this analysis, *F*(1, 98) = 12.80, *p* = 0.001, η*^2^_*part*_*_._ = 0.116. This indicates that the relation between sports affiliation and demand to compete while cooperating is independent from the relation of sports affiliation and mastery approach goals and conscientiousness.

### Discussion

Supporting Hypothesis 1, we found a clear difference in the demand to compete while cooperating with the same others (during practice or competitions) between team and individual sports. This difference also remains significant when statistically controlling for differences in mastery approach goals and conscientiousness between both athlete groups. As both athlete groups did not differ in other concepts related to cooperation and competition, team athletes are not more or less competitive or cooperative than individual athletes *per se*. However, supporting our rationale that the two sports affiliations differ in their relation between competing and cooperating, the results indicate that team athletes have to compete and cooperate with same others to a greater extent than individual athletes have to.

It should be noted that we used a newly developed scale that awaits construct validation, which might be considered as a limitation of the current study. At the same time, the face validity of the scale is very high – it asks for perceived demands and that is what we aimed to measure. Thus, the current study provides evidence that team sports lead to stronger perceived demands to compete while cooperating with the same others. A replication of the current study would nonetheless be beneficial.

## Study 2

The first study showed that team athletes (compared to individual athletes) perceive competing and, simultaneously, cooperating with the same others to be more of a requirement for their sport. This means that team athletes are less able to stop cooperating when competing with teammates or training partners than individual athletes. Study 2 examined whether, as a potential consequence of this demand, team athletes’ cognitive representation of competition excludes cooperating to a smaller extent than individual athletes’ representation of competition.

After inducing competition or a focus on intra-individual improvement (i.e., the control condition), we measured cooperative behavior (i.e., information sharing) in an unrelated subsequent task. This procedure allows to assess whether an activated concept (here: competition) (de)activates a certain behavioral strategy (here: cooperation). According to Hypothesis 2, individual athletes should show less cooperative behavior after a competition (compared to the control condition), whereas this effect should be smaller for team athletes. Evidence for this prediction would suggest that competition inhibits cooperating less among team athletes than among individual athletes.

For a subset of the participants in Study 2 we tested the cognitive association between competition and cooperation. These participants worked on a lexical decision task (LDT) with competition (vs. neutral) primes and cooperative (vs. neutral) targets, which assessed the accessibility of cooperative thoughts after priming competition (i.e., activating the representation of competition; for a review of facilitation and inhibition in LDT, see Neely, 1991; for an application to a social domain see Moskowitz et al., 2000). Support for Hypothesis 3 would be provided by a slowdown of responses to cooperative targets after competition primes by individual athletes, but less so by team athletes.

### Method

#### Participants

The data for Study 2 was collected on two occasions. Within the first occasion, 80 students from the university sports institute participated in return for a chocolate bar, a piece of fruit, and a beverage. In line with the procedure suggested by Bargh and Chartrand (2000), five participants who recognized a relation between the priming and the dependent variable in the funnel debriefing were excluded from the analysis. Including these participants did not alter the results substantially. Of the remaining 75 participants (46 women, age was assessed in categories: 22 participants were 18–21, 39 p. were 22–25, 13 p. were 26–30, and 2 p. were 31–40), 40 indicated to predominantly engage in individual sports (12 athletic sports/running, 5 swimming, 4 gymnastics, 3 tennis, 3 skiing, and 13 in other sports) and 35 in team sports (17 soccer, 8 handball, 6 volleyball, and 4 in other team sports).

The second data collection took place at the Olympic center (OSP) in Heidelberg, Germany. The 41 participants (16 women; *M*_age_ = 15.22, range = 14–16 years) were part of the national Olympic under-16 and under-15 teams. Because of the longer study duration due to the reaction time measure, the elite athletes received a higher compensation (€10, approximately US $11.5) as compared to the sports students. Seventeen engaged in an individual sport (12 of them swimming and 5 of them table tennis) and 24 in a team sport (basketball).

#### Procedure

Participants first signed an informed consent form; for the second data collection (at the OSP), we additionally obtained permission from the parentally authorized coaches of the youth athletes. Data collection in both occasions took place in a quiet room equipped with 20 laptops allowing the simultaneous participation of several athletes.

##### Reaction time measure

The assessment of the associative relation between competition and cooperation via a lexical decision task (LDT) with sequential priming was only taken by youth athletes at the OSP (at the beginning of the study). While doing so, participants were seated in front of a laptop in a seminar room of the OSP. The athletes of each youth squad (swimming male/female, table tennis male/female, basketball male/female) participated simultaneously (on four different days), because the time frame of the training camp only allowed a short period for the data collection.

In 72 critical trials, participants had to decide, as correctly and quickly as possible, whether or not a presented letter string represented an existing word or not (see Schvaneveldt et al., 1982). Half of the targets were non-words, the other half cooperation or control words. Each trial started with a fixation cross (400 ms). Then, competition or control words were presented as primes, followed by a black screen for 15 ms (stimulus onset asynchrony [SOA] = 200 ms), before the target appeared. After the decision on whether the target was an existing word (vs. not) was made, the screen went black for 500 ms before the next trial began. The order of trials was randomized, whereby this measure represented a 2 (prime: competition vs. control) × 2 (target: cooperation vs. control) *within-subject* design.

We included multiple competition primes [“schneller” (faster); “Wettkampf” (competition); “besser” (better); “behaupten” [to assert (oneself)]; “Konkurrenz” (rivalry); “durchsetzen” (to prevail)] and cooperation targets [“unterstützen” (to support); “zusammen” (together); “helfen” (to help); “vertrauen” (to trust); “gemeinsam” (collectively); “Teamgeist” (team spirit)] related to sports. We derived these words (i.e., primes) from interviews with individual and team sports experts (i.e., elite athletes and coaches) that we conducted in advance. We explicitly asked these experts to describe how they perceive “competition” as well as “cooperation” in their focal sport. In this way, we sought to identify primes that are able to activate the concept of competition on the one hand and cooperation on the other hand (or their meaning; cf. Simpson et al., 1989; Simpson and Kang, 1994).

##### Experimental manipulation

**FIGURE 1:**
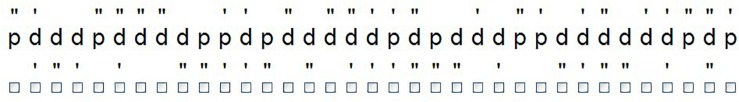
Example of a screen of the d2 test, comprising 35 letters that include 15 targets. Targets had to be marked by clicking the corresponding box.

For the main part of the study, individual and team athletes from both data collections worked on a cognitive achievement test (the d2 test of attention; see Brickenkamp and Zillmer, 1998) that served as the experimental manipulation. In our computer version of this test, 35 letters (d and p) with either one or two dashes above or below them were displayed horizontally on a screen for 15 s before the next screen appeared (see Figure 1). Only d letters with two dashes in total had to be marked. One test consists of five to ten screens. After the first test, the experimental manipulation was implemented as between-subjects factor.

Participants in the *competition condition* were informed that their performance in the next trials would be compared with the average performance of other students (university) or youth athletics (OSP) who also completed the test. Thus, their goal was to attain a better score in comparison with other members of their peer group (for a similar induction of competition, see Toma and Butera, 2009). Participants in the *control condition* were informed that their performance in the next trials would be compared with their own performance. Thus, their goal was to attain a better score in comparison with their own previous performance. Participants then completed the second run of the test.

##### Information sharing measure

Afterward, all participants worked on a riddle (the information pooling game by Steinel et al., 2010, Experiment 3) that was presented as being unrelated to the achievement task. Solving the riddle supposedly required sharing of some information with two fictitious others who also possessed information. Participants were displayed twelve pieces of information on one screen. To be able to solve the riddle, apparently at least eighteen pieces of information had to be exchanged by the supposed three persons. However, no goal instructions were given (e.g., whether the goal was to solve the riddle alone or together as a group; cf. Sassenberg et al., 2014). Instead, this goal ambiguity should allow the cognitive representation of the previous task to influence this subsequent task (also referred to as applicability; see for example Higgins and Brendl, 1995). Similar to a public goods dilemma, participants had to decide which of their twelve pieces of information they wanted to share correctly, to withhold, or to distort (i.e., to share the opposite in terms of content). Accordingly, we assumed the *number of correctly shared pieces of information* to constitute an indicator of cooperative behavioral tendencies, and thus added it as a dependent measure to the analysis.

After their information sharing decisions, participants actually did not receive information from others and did not have to further solve the riddle. Following the last run of the d2 test, participants at the university answered some exploratory and the demographic questions before they indicated their e-mail address for the debriefing mail on a separate sheet. In the university sample, we also tested the impact of sports affiliation on chronic performance-approach (three items, e.g., “It is important for me to do better than others”, α = 0.91) and mastery-approach goals (three items, e.g., “I want to learn as much as possible from intellectual tasks”, α = 0.72) adapted from Elliot and McGregor (2001) to “intellectual tasks” on a scale from 1 (*does not apply*) to 7 (*applies completely*). Participants were debriefed on the last screen. Finally, participants were thanked and compensated.

Importantly, the occasion of data collection did not moderate our predicted effect, *F*(1, 108) = 0.01, *p* = 0.913, η*^2^_*part*_*_._ < 0.001. Therefore, we deemed it justified to combine the information sharing measure from both data collection occasions to reach an adequate statistical power for the 2-by-2 design regarding this dependent measure.

### Data Analysis

#### Information Sharing

To test Hypothesis 2 we conducted an ANOVA with the factors condition (competition vs. control) and sports affiliation (individual vs. team) and the number of correctly shared pieces of information as dependent variable. In particular, Hypothesis 2 implied that individual athletes in the competition condition subsequently shared less information compared to all the other conditions (individual athletes in the control condition as well as team athletes in either the competition or the control condition). This prediction was tested with a contrast (−3 + 1 + 1 + 1). Given that the information sharing indicator was not normally distributed (Shapiro–Wilk test: *p* < 0.001), we also computed a (non-parametric) Mann–Whitney Test resembling this contrast.

#### Response Time Data From the LDT

Before aggregating the response time data, latencies from false answers and from answers faster than 300 ms were excluded. Responses were cut off at 3 s to control for outliers (for similar cut-off values examining children with the LDT, see for example Ratcliff et al., 2012; Van den Boer et al., 2012). Two participants with an error rate of more than 20% were excluded from this analysis (*M*_error rate_ = 4.67%). To estimate the reported effect sizes regarding this measure, we calculated a difference score based on the two within-factors (Difference Score = [RT for competition prime | cooperation target – RT for control prime | cooperation target] – [RT for competition prime | control target – RT for control prime | control target]).

Hypothesis 3 predicted that competition (vs. control) primes would slow down reactions to cooperation (vs. control) targets in the LDT of Study 2b for individual, but not for team athletes. To test this prediction, we entered the reaction times for target words (in milliseconds) into a 2 (sports affiliation: individual vs. team) × 2 (prime: competition vs. control) × 2 (target: cooperation vs. control) mixed ANOVA with *repeated* measures on the last two factors. As frequently observed in reaction time tasks, response latencies were not normally distributed (Shapiro–Wilk test: all *p*s < 0.01). Therefore, we also computed a Mann–Whitney Test comparing the Difference Score mentioned above between the two sports affiliation conditions as a non-parametric test of the predicted effect.

In an additional analysis, we tested whether the relation between mastery approach (but not performance approach) goals and sports affiliation could be replicated. To this end, two separate ANOVAs with condition (competition vs. control) and sports affiliation (individual vs. team) as independent variables and the respective goal type as dependent variables were conducted. Given that both scales were not normally distributed (Shapiro–Wilk test: both *p*s < 0.005), we also computed a (non-parametric) Mann–Whitney Test examining the relation between sports affiliation and both types of achievement goals.

### Results

#### Information Sharing

**FIGURE 2:**
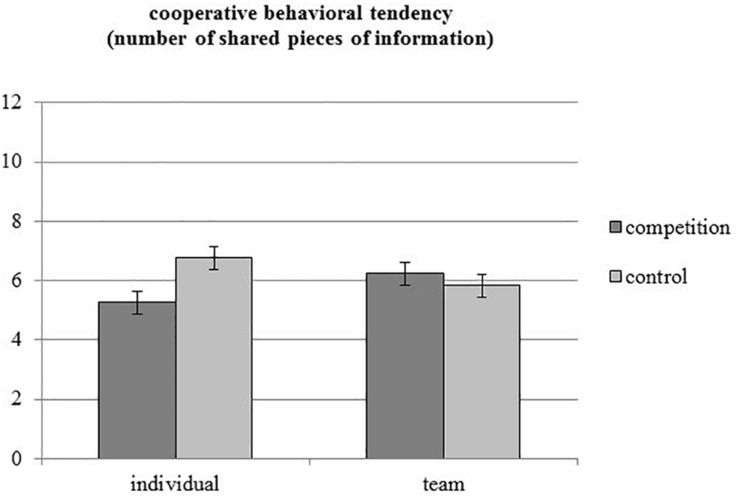
Number of shared pieces of information with uninvolved others by individual versus team athletes in the competition versus control condition (Study 2, *N* = 116). Error bars denote one standard error around the mean.

Hypothesis 2 predicted that competition (vs. control) subsequently reduced information sharing for individual, but not for team athletes. The predicted interaction was significant, *F*(1, 112) = 6.17, *p* = 0.014, η*^2^_*part*_*_._ = 0.052 (see Figure 2). In line with Hypothesis 2, individual athletes in the competition condition shared less information compared to all the other conditions, *F*(1, 112) = 5.17, *p* = 0.025, η*^2^_*part*_*_._ = 0.044, *MD* = 1.023, CI_95__%_ = [0.131, 1.915]. This was confirmed by the non-parametrical Mann–Whitney test, *U* = 877, *z* = 2.32, *p* = 0.020.

Simple comparisons further showed that individual athletes shared less information with uninvolved others in the competition condition (*M* = 5.25, *SE* = 0.39) than in the control condition (*M* = 6.76, *SE* = 0.39), *F*(1, 112) = 7.53, *p* = 0.007, η*^2^_*part*_*_._ = 0.063, *MD* = 1.509, CI_95__%_ = [0.420, 2.598]. Team athletes, however, shared as much information in the competition condition (*M* = 6.23, *SE* = 0.38) as in the control condition (*M* = 5.83, *SE* = 0.39), *F*(1, 112) = 0.56, *p* = 0.454, η*^2^_*part*_*_._ = 0.005, *MD* = −0.406, CI_95__%_ = [−1.476, 0.665]. Thus, supporting Hypothesis 2, only individual but not team athletes demonstrated a carry-over effect of competition on spontaneous information sharing and therefore a reduction of cooperative behavioral tendencies following competition.

#### Reaction Times in the LDT

Hypothesis 3 predicted that competition (vs. control) primes slowed down reactions to cooperation (vs. control) targets in the LDT for individual, but not for team athletes. Results revealed a main effect of sports affiliation, *F*(1, 37) = 4.55, *p* = 0.040, η*^2^_*part*_*_._ = 0.109, that was qualified by the predicted three-way interaction between sports affiliation (i.e., the between factor), prime and target, *F*(1, 37) = 5.11, *p* = 0.030, η*^2^_*part*_*_._ = 0.121, *MD* = 61.66 ms, CI_95__%_ = [6.41, 116.90]. A non-parametric test likewise provided support for Hypothesis 3 (Mann–Whitney *U* = 91, *z* = 2.57, *p* = 0.010 for the Difference Score).

**FIGURE 3:**
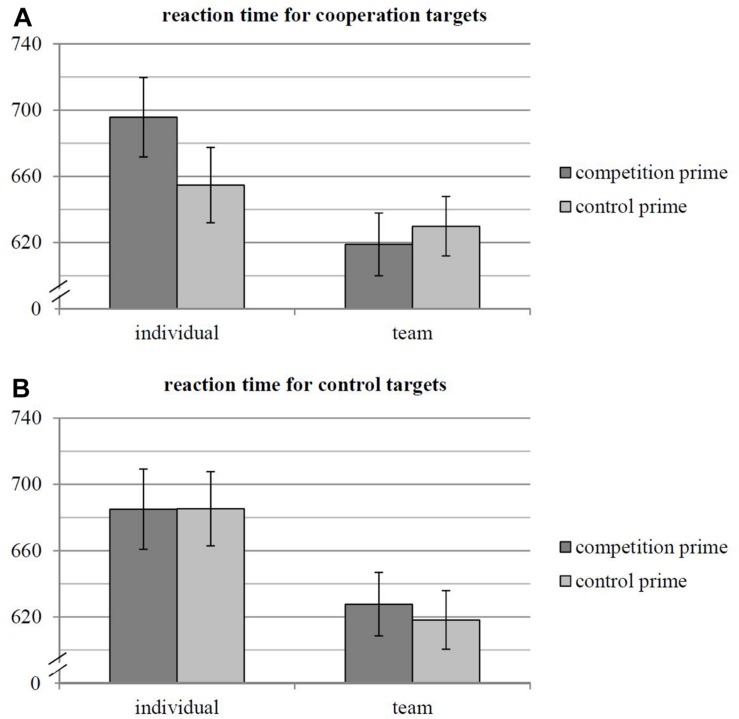
**(A,B)** Mean reaction times in milliseconds to cooperation targets (**A**, top) and to control targets (**B**, bottom) for individual versus team athletes in trials with either competition or control primes (only youth athletes at the OSP, *N* = 39, two between conditions). Error bars denote one standard error around the mean.

As can be seen in Figure 3A, individual athletes reacted slower to *cooperation targets* after competition primes (*M* = 695.65 ms, *SE* = 23.96 ms) compared to control primes (*M* = 654.71 ms, *SE* = 22.71 ms), *F*(1, 37) = 3.87, *p* = 0.057, η*^2^_*part*_*_._ = 0.095, *MD* = 40.94 ms, CI_95__%_ = [−1.24, 83.12]. In contrast, team athletes did not show such a difference in RT for *cooperation targets* following competition vs. control primes (*M* = 618.90 ms, *SE* = 18.94 ms vs. *M* = 629.85 ms, *SE* = 17.95 ms), *F*(1, 37) = 0.44, *p* = 0.510, η*^2^_*part*_*_._ = 0.012, *MD* = −10.94 ms, CI_95__%_ = [−44.29, 22.40]. Beyond that, there was no difference between the two prime categories in the reaction to *control targets*, neither in individual nor in team athletes, both *F*s < 0.6, *p*s > 0.4, η*^2^_*part*_*_._s < 0.02 (see Figure 3B). This suggests that, for individual athletes, competition and cooperation are also incompatible on the semantic level. However, team athletes seem to cognitively reconcile both allegedly incompatible concepts. These findings support Hypothesis 3.

#### Additional Analysis

Neither a difference in conditions, nor between individual and team athletes, nor an interaction occurred for both types of achievement goals (i.e., mastery approach and performance approach goals), all *F*s < 1.8, *p*s > 0.150, η*^2^_*part*_*_._s < 0.03; non-parametric Mann–Whitney Test: both | *z*| < 0.5, both *p*s > 0.6. Hence, we did not replicate the effect found in Study 1 (with a different measure). Further research is needed to decide whether mastery approach goals are related to sports affiliation.

### Discussion

The results of Study 2 demonstrate that cooperative behavior (i.e., information sharing with uninvolved others) is undermined after experiencing competition for individual athletes, but less so for team athletes (Hypothesis 2). In addition, athletes reacted slower to cooperative targets after competition compared to control primes when engaging in individual sports, but less so when engaging in team sports (Hypothesis 3). Taken together, these findings suggest that team (compared to individual) athletes’ cognitive representation of competition does not necessarily exclude cooperating. One limitation of this study is that we did not include a manipulation check for the competition manipulation. However, given that the expected effects of competition occurred in the individual sports condition, we deem it appropriate to assume that the manipulation was successful.

## General Discussion

The current research examined if engagement in team versus individual sports moderates the incompatibility of competition and cooperation. Study 1 indicated that team athletes, indeed, attribute a higher demand to compete and to cooperate with the same others to their focal sport than individual athletes attribute to their sport. Aside from this, barely any differences associated with sports affiliation where found. Based on the higher demand to cooperate and compete with the same others, we expected team athletes to have more cooperative behavioral tendencies and cooperative thoughts than individual athletes (only) when experiencing competition. This would indicate a representation of competition that also allows cooperating to a certain extent—which is what team athletes are required to do on a regular basis. Study 2 showed that individual athletes spontaneously reduced information sharing (i.e., a cooperative behavioral tendency) with uninvolved others subsequently to a competition (vs. control). However, team athletes did not show this carry-over effect of competition. In addition, individual athletes reacted more slowly to cooperative targets in trials with sequential competition (vs. control) primes, whereas team athletes did not show such an inhibitory relation. Taken together, these findings demonstrate that for team athletes, competition neither reduces cooperative behavior, nor inhibits cooperative thoughts. Therefore, the present research suggests that for team athletes, competing with others does not necessarily exclude cooperating with them.

Sport psychological research comparing team and individual athletes barely revealed any personality differences between these athletic groups (see Allen et al., 2011). Intuitively, one might think that they should differ on dimensions that are related to social aspects of personality, since team athletes have to interact with their teammates. However, research suggests that, similar to team athletes, competitive individual athletes rarely practice on their own (Evans et al., 2012). This may be the reason why there are no differences between competitive individual and team athletes in overall prosocial or assertive characteristics that would be relevant for an applied setting also beyond sports, for example personnel selection (see Tanguay et al., 2012). In fact, research in organizational psychology dealing with interpersonal differences between team and individual sports did not reveal clear differences in social skills so far that would render this distinction actually relevant for human resources departments (see Kanning and Kappelhoff, 2012). Study 1 underlines this assumption by showing no differences in social value orientation, social comparison orientation, or competitive goals. However, this study also demonstrated that the *social context* of team and individual sports is perceived differently: Team sports demand competition as well as cooperation with the same others during performance to a greater extent than individual sports. Therefore, a crucial difference between team and individual athletes might lie in their *cognitive relation* between competitive and cooperative behavior. Supporting this hypothesis, Study 2 revealed that competition diminishes cooperative behavioral tendencies as well as cooperative thoughts for individual athletes to a stronger extent than for team athletes. In sum, the current research makes an important contribution to (a) research examining interindividual differences between sports affiliations and (b) research examining the (negative) impact of competition by including sports affiliation as an individual characteristic and by incorporating competition as a situational dimension (cf., Mischel and Shoda, 2010).

For individual athletes, we replicated and extended previous findings. For example, going beyond research showing that prosocial tasks *enhance* the accessibility of cooperative thoughts (Greitemeyer and Osswald, 2011), the results of the LDT in Study 2 demonstrate that previous activation of competition can systematically *decrease* the accessibility of cooperative thoughts. Consequently, social cues not only have the potential to enhance the accessibility of concepts within the cognitive network, but also to inhibit potentially interfering concepts. In line with the carry-over effect of competition on the perception of uninvolved others (Sassenberg et al., 2007), we further showed that competition potentially also reduces cooperative behavior (i.e., information sharing) during *subsequent*, unrelated tasks. This might have crucial implications for sports or other teams in which members have to work together and rely on each other’s knowledge. Team members who are competing (e.g., for a starting position or a promotion) could reduce task-related information exchange or other necessary cooperation, even when the recipients are not involved in that particular competition (e.g., teammates on different positions or colleagues of another working unit). Such an impairment of knowledge exchange outside the competition thus not only poses a threat to the effective cooperation between competitors, but also to the success of the whole team.

### Limitations

The focus of the current research was to differentiate between team and individual sports on a general level. One limitation of our studies is that we did not further differentiate within team as well as within individual sports. Thus, we cannot rule out that some of our individual athletes also engaged in more cooperative forms of their sports from time to time, for example doubles in tennis, table tennis or badminton. To some extent, we believe that doubles are behaviorally interdependent during the performance. For example, table tennis players have to step aside for the other player to hit the ball. Such forms of behavioral interdependence should weaken the dissociation between competition and cooperation for respective individual athletes. When isolating individual athletes who engage in doubles (and thus converge to the team sports sample), the difference between team and individual athletes regarding the dissociation between competition and cooperation might be even stronger than we found in our studies.

In study 2, only youth athletes at the Olympic center fulfilled the lexical decision task. The reason is that we wanted to add a classical association measure after occasion one (University sub-sample) in order to broaden our methodological approach. Of course, the findings would have been stronger when based on a bigger athlete sample. Nevertheless, a *post hoc* power analysis revealed a medium observed power of 0.69 for our main analyses (for Study 1,Study 2, and for the subset of participants at the OSP in Study 2), which is only slightly below a power we were initially aiming at (0.80).

### Future Research

The incompatibility of competition and cooperative behavior seemingly does not pertain to team athletes who have to compete as well as cooperate with one and the same teammates. This twofold demand is comparable to what the management literature describes as *co-opetition*, a term originally implying simultaneous cooperation and competition between firms (Nalebuff and Brandenburger, 1996). Only recently, Galinsky and Schweitzer (2015) published a book in which they also point out the prevalence of co-opetition in everyday life by arguing that social relationships often contain competitive *as well as* cooperative aspects (i.e., others are always “friends & foes”; for co-opetition within organizations see for example Tsai, 2002). Thereby, co-opetition fundamentally differs from social interdependent situations that have received most attention in psychological research. First, it differs from intergroup competition, because here individuals compete with other teams and they cooperate within their own team. Thus, intergroup competition – a situation that team athletes encounter frequently (*as do individual athletes*; cf. Evans et al., 2012) – does *not* require reconciling competition and cooperation with the *same target*. This might be the reason why intergroup competition has been shown to reduce cooperative intentions similarly to or even stronger than interpersonal competition (e.g., Sherif, 1966; Scheepers et al., 2003). Second, competition and cooperation with the same target also differs from usual social dilemmas examined in mixed-motive interdependence research: In contrast to handling a dilemma, members of a sports team, exemplarily, cannot choose to either compete *or* to cooperate, but need to show both behaviors simultaneously or in close succession to be successful. Recently, the need to reconcile these demands has been shown to enhance cognitive flexibility (Landkammer and Sassenberg, 2016). At least, the current research considers the possibility that being confronted with this need for reconciliation on a frequent basis (as team athletes are) diminishes the psychological contrast between competing and cooperating. Following this reasoning, it would be very interesting to examine team athletes’ cognitive flexibility subsequent to experimentally induced co-opetition situations. We would assume that for them, cognitive flexibility should not be enhanced as much, because the competitive demand and the cooperative demand should be perceived as less conflicting.

An important question is whether athletes self-select their sports affiliation based on the different demands in team versus individual sports, or whether team athletes learn to reconcile competitive and cooperative behavior through engagement in their sport. Athletes in our studies were predominantly engaging in competitive sport, some of which even belonged to the national Olympic youth squad, implying a high number of practice sessions. This serious engagement hints toward a relation between the continuous *experience* of co-opetition and the cognitive reconciliation of competition and cooperative behavior. The lack of other chronic differences between sports affiliations related to competition and cooperation (as in the Preliminary Study) points to this assumption – as well as do cultural comparisons between eastern and western societies. According to Fülöp (2009), Japan is a competitive-cooperative society with a highly competitive school system but, at the same time, Japanese are continuously required to cooperate with others. Probably as a result of this socialization, Japanese conceptualize opponents as partners who take on the cooperative function of improving each other during the competition. Instead, the more common perception in western countries is that the rivals merely have to be defeated (Fülöp, 2009).

It is necessary and, from our point of view, very desirable to longitudinally examine whether engagement – or *socialization* – in team sport actually leads to a change in the relationship between competition and cooperative behavior. If one assumes this causal order, an interesting implication of our findings would be that engagement in (team) sports is able to change individual characteristics in a positive way (also called the change hypothesis; see Allen et al., 2013). In this vein, it might also be interesting to compare casual and competitive (team) sport samples. Therefore, the current research contributes to keeping the discussion about the societal impact of sports and transfer effects alive.

### Conclusion

To conclude, by using different sports affiliations, the current research demonstrated that competition and cooperation are not necessarily incompatible psychological concepts. Instead, team athletes seem to be able to reconcile competition and cooperative behavior. In this sense, “team player skills” could be operationalized as the ability to compete with team members while, at the same time, not ceasing to cooperate with them.

## Data Availability Statement

The datasets generated for this study are available on request to the corresponding author.

## Ethics Statement

In order to recruit adult athletes, data collection was scheduled for a specific time frame at a university sports facility, in which we aimed to recruit as many participants from this specific sample as possible. This procedure of recruitment was identical across Study 1 and the University sample of Study 2. Ethical approval for the current studies was provided by the institutional review board (Lokale Ethikkommission) of the Leibniz-Institut für Wissensmedien (Tübingen, Germany; LEK 2011/013). The inclusion of the non-adult sample in Study 2 was additionally approved by the Olympic Sports Center Rhein-Neckar (Heidelberg, Germany). Written informed consent was obtained from all adult participants as well as from the respective coaches of the Olympic Sports Center (OSP). According to German law the OSP was able to provide (and provided) informed consent for the youth athletes as it has supervision duty.

## Author Contributions

All authors listed have made a substantial, direct and intellectual contribution to the work, and approved it for publication.

## Conflict of Interest

The authors declare that the research was conducted in the absence of any commercial or financial relationships that could be construed as a potential conflict of interest.

## Footnotes

1 Degrees of freedom vary due to missing data.

## Appendix

Items that measured the perceived demand to compete and cooperate with same others in the Preliminary Study (English translation; Original Version in German see below):

1. In order to be successful at my sport, you often have to prevail against the same people you cooperate with.
2. My relationships with the athletes that I practice with are likewise competitive as well as cooperative.
3. When I practice together with others, cooperation is at least as important as the competition among us.
4. My coach monitors both my individual performance compared to others and my cooperation with them during practice.
5. In my sport it is important to demonstrate your superiority in certain areas in comparison to others with whom you share the same goal.
6. During practice I try to outperform others, although the actual goal is that we mutually improve ourselves.
7. The team spirit does not suffer during practice when single team members compete with each other.
8. In my sport, the achievement of common goals is similarly important as the achievement of individual goals.
9. My coach expects me to be better than others and simultaneously behave as a team player.
10. My practice together with others is concurrently characterized by team spirit and rivalry.

Original Items in German:

1. Um in meinem Sport erfolgreich zu sein, muss man sich häufig gegen die gleichen Personen durchsetzen, mit denen man auch zusammenarbeitet.
2. Meine Beziehungen zu den Sportlern, mit denen ich trainiere, sind gleichermaßen kompetitiv wie auch kooperativ.
3. Wenn ich mit anderen trainiere ist Kooperation mindestens genauso wichtig wie der Wettbewerb untereinander.
4. Mein Trainer/meine Trainerin beobachtet im Training sowohl meine individuellen Leistungen im Vergleich zu anderen als auch meine Zusammenarbeit mit ihnen.
5. In meinem Sport ist es wichtig zu demonstrieren, dass man in bestimmten Bereichen besser ist als andere, mit denen man am selben Strang zieht.
6. Im Training versuche ich besser zu sein als die anderen, obgleich das eigentliche Ziel ist, dass wir uns alle gemeinsam verbessern.
7. Der Teamgeist leidet während des Trainings nicht darunter, wenn einzelne Gruppenmitglieder miteinander konkurrieren.
8. Das Erreichen gemeinsamer Ziele ist in meinem Sport ebenso wichtig wie das Erreichen individueller Ziele.
9. Mein Trainer/meine Trainerin erwartet, dass ich andere übertreffe und mich gleichzeitig wie ein Teamplayer verhalte.
10. Mein Training mit anderen ist gleichzeitig geprägt von Teamgeist und Konkurrenzkampf.

Items of the Personality measure applied in Study 1 (Rammstedt and John, 2005; translated from German):

*Extraversion:* I am reticent. (reversed) I am enthusiastic and able to enthrall others. I am a quite person. (reversed) I am sociable.

*Agreeableness:* I tend to criticize others. (reversed) I easily trust others. I can be cold and distant. (reversed) Sometimes I behave shrugged and repellent toward others. (reversed)

*Conscientiousness:*I complete my tasks thoroughly. I am comfortable and inclined to laziness. (reversed) I am proficient and work fast. I make plans and carry them out.

*Neuroticism:* I get easily dejected and depressed. I am relaxed and hard to be stressed. (reversed) I worry a lot. I often get a little nervous and insecure.

*Openness:* I appreciate artistic and esthetic impressions. I am interested in many things. I like to think profoundly about things. I am imaginative and have many fantasies. I am little interested in arts. (reversed)
